# Hepatocellular Carcinoma Occurrence and Recurrence in Hepatitis C-infected Patients Treated with Direct-acting Antivirals

**DOI:** 10.7759/cureus.2843

**Published:** 2018-06-19

**Authors:** Taseen Syed, Javid Fazili, Ijlal Akbar Ali, Daniel Zhao, Diane Hughes, Sultan Mahmood

**Affiliations:** 1 Internal Medicine, University of Oklahoma Health Sciences Center, Oklahoma City, USA; 2 Section of Digestive Diseases & Nutrition, University of Oklahoma Health Sciences Center, Oklahoma City, USA; 3 Section of Digestive Diseases and Nutrition, University of Oklahoma Health Sciences Center, Oklahoma City, USA; 4 Department of Biostatistics, University of Oklahoma Health Sciences Center, Oklahoma City, USA; 5 Gastroenterology Fellow Section of Digestive Diseases & Nutrition, University of Oklahoma Health Sciences Center, Oklahoma City, USA

**Keywords:** hepatitis c, direct-acting antivirals (daa), sustained virological response

## Abstract

Introduction

Multiple studies have shown the efficacy of the new direct-acting antivirals (DAAs) with a cure rate of over 90% in hepatitis C virus (HCV)-infected patients. Some recently published studies have suggested an increased incidence of de novo and recurrent hepatocellular carcinoma (HCC) in cirrhotic patients in sustained virological response (SVR) after completing therapy. A possible mechanism is the breakdown of immune surveillance after starting DAAs. We report a retrospective analysis on a population of chronic HCV infected patients, with and without a prior history of HCC, who developed HCC after receiving DAAs in the hope of adding to existing literature and in pursuit of greater clarity into this emerging concern with DAAs. Methods

We analyzed 497 HCV-infected patients who were treated with DAAs, or a combination of DAA with interferon, from January 2014 to April 2017 at the Veterans Medical Center, Oklahoma City. Descriptive analysis, including the mean and standard deviation for different variables, was used. The cohort was divided into two groups: cirrhotic and non-cirrhotic. The analysis was run in the cirrhotic group between the subgroups who developed HCC and who did not. Results

Data from a total of 233 cirrhotic patients were analyzed. We further subdivided these patients into those who eventually were diagnosed with HCC (group 1) and those who were not (group 2). These subgroups were comparable in regards to race, gender, baseline serum aspartate aminotransferase (AST), alanine aminotransferase (ALT), platelets, sodium, HCV genotypes, and pretreatment viral load. All patients completed therapy. The rate of SVR was much lower in group 1 compared to group 2 (62.5% vs 88.94%, p = 0.002), respectively. Model End-stage Liver Disease (MELD) score, Child-Turcotte-Pugh (CTP) score, and Fibrosis-4 (FIB-4) score were higher in the group that developed HCC. The average time period (weeks) from DAA therapy to HCC diagnosis was 48.2 weeks. The remaining 264 non-cirrhotic patients had no reported cases of HCC. Conclusion

From a total of 497 treated HCV-infected patients, 233 (46.88 %) had cirrhosis, out of which 16 (6.86%) were reported to develop HCC during or after DAA therapy was initiated. The remaining 217 (93.1%) cirrhotic patients did not develop HCC. As per our comparison, achieving SVR in cirrhotic patients should not preclude HCC screening, and more studies are needed to assess the risk of HCC in patients who achieve SVR but have a high FIB-4 score. In fact, patients who do not achieve SVR may be at a higher risk of eventually developing HCC and may be candidates for closer surveillance.

## Introduction

Multiple recent studies have suggested an increased incidence of de novo and recurrent hepatocellular carcinoma (HCC) in cirrhotic patients with a sustained virologic response (SVR) after completing therapy with direct-acting antivirals (DAAs) [1-4]. Recent studies also suggest lower SVR rates in patients with hepatitis C virus (HCV)-related cirrhosis and underlying HCC when compared to patients with only HCV cirrhosis [5]. In the hope of adding to the literature, we performed a retrospective analysis to assess the rates of SVR and the incidence and recurrence of HCC in chronic HCV-infected patients who were treated with DAAs at our institution.

## Materials and methods

We analyzed 497 HCV-infected patients who were treated with DAAs, or a combination of DAA with interferon, from January 2014 to April 2017 at the Veterans Medical Center, Oklahoma City. Descriptive analysis, including the mean and standard deviation for different variables, was used. The cohort was divided into two groups: cirrhotic and non-cirrhotic. The analysis was run in the cirrhotic group between the subgroups who developed HCC and who did not. ​​​​​​​A P value < 0.05 was considered statistically significant.

## Results

Two hundred and thirty-three patients (46.88 %) had cirrhosis, of which 16 (6.86%) developed HCC (11 de novo and five recurrent cases) during or after DAA therapy was initiated, whilst the remaining 217 (93.1%) cirrhotic patients did not develop HCC. None of the non-cirrhotic patients developed HCC. We further subdivided the cirrhotic group into those who eventually were diagnosed with HCC (group 1) and those who were not (group 2). These subgroups were comparable in regards to race, gender, baseline serum aspartate aminotransferase (AST), alanine aminotransferase (ALT), platelets, sodium, HCV genotypes, and pretreatment viral load. All patients completed therapy. The rate of SVR was much lower in group 1 compared to group 2 (62.5% vs 88.94%, p = 0.002), respectively. The Fibrosis-4 (FIB-4) score was higher in group 1 as compared to group 2 (7.88 vs 4.09, p = 0.048), respectively. The average time period (weeks) from completion of DAA therapy to HCC diagnosis was 48.2 weeks and from time of SVR to HCC detection was 33.3 weeks (Table 1).

**Table 1:** Table: Comparative analysis between cirrhotic patients with and without hepatocellular carcinoma. Demographic and laboratory variables along with outcome assessment with Chi-Square Test or T-test. Acronyms: Hepatocellular carcinoma (HCC), Hepatitis C Virus (HCV), Aferican American (AA), Model for End-Stage Liver Disease (MELD), Child-Turcotte-Pugh (CTP), Fibrosis-4 (FIB-4) Score, sustained virological response (SVR), Interfron (IFN), and direct acting antiviral (DAA).

Variables	HCV treated cirrhotic patients with HCC (n = 16)	HCV treated cirrhotic patients without HCC (n = 217)	P-Value
AGE, average	66.19	62.09	0.005
Male sex, n (%) [number & percentage of patients]	16 (100)	212 (97.7)	0.539
RACE			
a. White, n (%)	12 (75)	155 (71.43)	0.913
b. AA, n (%)	2 (12.5)	26 (11.98)	
c. Other, n (%)	2 (12.5)	36 (16.59)	
HCV Genotype			0.624
a. Genotype 1a	9 (56.25)	108 (49.76)	
b. Genotype 1b	3 (18.75)	44 (20.27)	
c. Genotype 2	2 (12.5)	46 (21.19)	
d. Genotype 3	2 (12.5)	2 (0.92)	
History of alcohol, n (%)	10 (62.5)	212 (97.69)	<0.001
History of smoking, n (%)	11 (68.75)	181 (83.4)	0.113
History of IV drug use, n (%)	5 (31.25)	139(64)	<0.001
Pre-Treatment Viral Load (IU/ml) Mean Value	1,286,176	4,484,995	0.084
Patients achieving SVR, n (%)	10 (62.5)	193 (88.94)	0.002
MELD Score, mean (median) at SVR	11.31(11)	9.88 (9)	0.173
CTP Score at SVR, mean (median)	6.31(6)	5.73 (5)	0.064
FIB-4 Score at SVR, mean (median)	7.88 (6.52)	4.98 (2.96)	0.048
AST at SVR, mean (median)	62.81 (48)	40.33 (28)	0.104
ALT at SVR, mean (median)	45.75 (29)	31.55 (23)	0.201
Hemoglobin at SVR, mean (median)	13.06 (13.05)	14.09 (14.5)	0.045
T. Bilirubin at SVR, mean (median)	1.53 (1.3)	1.03 (0.8)	0.005
Albumin at SVR, mean (median)	3.23 (3.2)	3.73 (3.8)	<0.001
Na at SVR	136.69 (137)	142.04 (137)	0.758
Platelets at SVR	108.75(96.5)	132.84(130.5)	0.173
Treatment naïve/Non-Treatment Naïve, n (%)			0.005
a. Treatment naïve, n (%)	6 (37.5)	155 (71.43)	
b. Non-Treatment naïve, n (%)	10 (62.5)	62 (28.57)	
Treatment regimen, n (%)			
a. DAA only based regimen, n (%)	13 (81.25)	207 (95.39)	
b. DAA & IFN based regimens, n (%)	3 (18.75)	10 (4.6)	
c. Use of Ribavirin, n (%)	12 (75)	115 (52.99)	
Comorbidities due to Chronic Liver Disease			
a. Ascites, n (%)	2 (12.5)	14 (6.48)	0.359
b. Esophageal Varices, n (%)	8 (50)	47 (21.66)	0.01

## Discussion

According to the Centers for Disease Control (CDC), of patients infected with hepatitis C, 5% - 20% develop cirrhosis over 20-30 years. Of these, 1% - 5 % die due to decompensated cirrhosis or HCC. Unadjusted data from a study conducted over a course of a decade (2001 - 2013) in United States veterans, published in November 2015, showed an incidence of HCC increased by 265%, mortality in cirrhotics increased by 51% and by 285% in patients with HCC [6]. With this global burden of hepatitis C, DAA-based regimens, with high efficacy and optimal safety profiles, are the best treatment option to date. However, recent studies have questioned the safety profile of these DAAs [2, 7-8]. A proposed hypothesis is the disruption of immune surveillance after starting DAA-based antiviral therapy [3]. At the other end of the spectrum, analysis of three French prospective multicenter cohorts, a prospective observational study, and analysis of a retrospective cohort of HCV-infected veterans treated with DAAs all showed no increase in HCC recurrence in patients treated with DAAs [4, 9-10]. Recent studies also demonstrate a lower SVR in HCV cirrhotics with underlying HCC. There are numerous proposed mechanisms for this, including HCC acting as a reservoir for HCV replication, distorted liver architecture due to HCC, lack of delivery of DAAs into HCC, different resistant strains of HCV infected patients with HCC, and immune dysregulation [5].

## Conclusions

As per our comparison, achieving SVR in cirrhotic patients should not preclude HCC screening and more studies are needed to assess the risk of HCC in patients who achieve SVR but have a high FIB-4 Score. In fact, patients who do not achieve SVR, may be at a higher risk of eventually developing HCC, and may be candidates for closer surveillance. Affirmative conclusions cannot be drawn based on the nature of all studies to date but it reinforces the need for statistically stronger studies on larger cohorts that takes into account all the flaws of previous studies. In this current era of questionable association of DAAs with HCC, ongoing HCC surveillance in cirrhotics achieving SVR from DAAs is a reasonable approach until we get further clarification.

This work was presented as poster presentation at Digestive Disease Week 2018.
